# Quantum-enhanced reconfigurable in-memory stochastic computing

**DOI:** 10.1038/s41377-025-02181-6

**Published:** 2026-03-18

**Authors:** Hong-Zhe Yang, Jian-Peng Dou, Feng Lu, Xiao-Wen Shang, Chao-Ni Zhang, Heng Zhou, Hao Tang, Xian-Min Jin

**Affiliations:** 1 Center for Integrated Quantum Information Technologies (IQIT), School of Physics and Astronomy and State Key Laboratory of Photonics and Communications, Shanghai Jiao Tong University, Shanghai, China; 2 Hefei National Laboratory, Hefei, China; 3 TuringQ Co. Ltd., Shanghai, China; 4 Chip Hub for Integrated Photonics Xplore (CHIPX), Shanghai Jiao Tong University, Wuxi, China

**Keywords:** Quantum optics, Optical data storage

## Abstract

In-memory computing, which enables computation directly within memory, represents an efficient approach to processing massively parallel computation tasks that are intractable for conventional computers. However, implementations of in-memory computing have been primarily limited to the classical regime, with its nonclassical counterpart yet to be fully explored. Quantum memory, with its unique capability to generate, preserve, and nontrivially operate on quantum states, offers spectacular quantum-enhanced advantages and is thus a promising candidate for in-memory computing. Here, leveraging a room-temperature quantum memory, we demonstrate a quantum-enhanced and reconfigurable in-memory stochastic computing system, where correlated photons, randomly produced in the quantum memory, serve as the computing resources. We show that addition and multiplication operations can be straightforwardly achieved by accumulating photon counts, and multiple computing tasks can be accelerated by mapping them into parallel accumulations of photon counts. Furthermore, the calculation results are obtained through stochastic processes, ensuring security in remote computation since no efficient information can be distinguished by eavesdropping on a small portion of the computation data. This in-memory computing system is enhanced by nonclassical correlations, which accelerate computing process and may stimulate future research and applications in the emerging field of quantum-enhanced computing architectures.

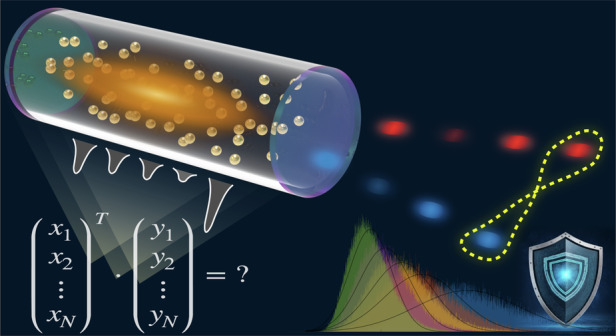

## Abstract (web summary)

Quantum memory — with its unique ability to generate, preserve, and perform nontrivial operations on quantum states — provides powerful quantum-enhanced capabilities and is a promising platform for in-memory computing.

## Introduction

With the rise of artificial intelligence alongside large amounts of data, the demand for efficiently processing massively parallel computation tasks becomes progressively prominent. However, it is inaccessible or challenging for conventional computers to meet this demand, due to intolerable energy cost and computing latency incurred by processor-memory dichotomy^1,2^. This motivates researches on new computing architectures. Non-von Neumann architecture, accompanied by advances of integrated photonic chips^3–7^, is proposed to efficiently process conventionally intractable tasks in a way of physical-layer parallel computation^8–14^. As one promising non-von Neumann architecture, in-memory computing breaks the traditional concept of separate processing and memory units^15–17^, and it even no longer requires deterministic storage, i.e., memory devices can be implemented in a way of stochastic computing^18–21^. Up to now, based on various memory mechanisms and materials, the implementations of in-memory computing have been realized in different systems ranging from electronic^22–27^ to photonic platforms^5,28–32^, and in-memory computing has been commonly recognized as a promising solution for efficiently processing massively parallel data^33–38^, such as applications in accelerating matrix-vector multiplication. Section SA of the Supplementary Information provides a comprehensive introduction to the foundational concepts and key challenges in stochastic computing, in-memory computing, and quantum computing.

Despite significant advances in in-memory computing, these achievements typically rely on the macroscopic properties of memory units in a classical manner, such as the resistance of memristive memory or the transmittance of non-volatile phase-change memory. To date, a memory system that operates intrinsically in the nonclassical regime has not been explored for demonstrating in-memory computing. Quantum memory^39,40^, capable of preserving quantum states, plays a crucial role for scalable quantum technologies which promise to outperform their classical counterparts^41–46^. Over the last 20 years, a tremendous amount of work on quantum memory has been done based on various protocols, such as electromagnetically induced transparency^47–51^, Raman memory^52–55^, photon-echo memory^56–59^, Duan-Lukin-Cirac-Zoller (DLCZ) protocol^60–63^, off-resonant ladder (or cascaded) memory^64,65^, optical loop memory^66,67^, and so on. Besides, the wide applications of quantum memory include long-distance quantum communication^68–75^, multiphoton synchronization^65,66,76^, hybrid quantum networks^67^ and single-photon sources^77–79^. Considerable efforts have been dedicated to make quantum memory practical for these suggested applications mentioned above. However, efforts dedicated to a full exploitation of the significance of quantum memories, such as those for constructing unconventional computing, are not enough, although applications of quantum memory are promising.

Here, leveraging a room-temperature quantum memory, we demonstrate an in-memory stochastic computing system that is intrinsically reconfigurable for various computing tasks, due to its controllable and programmable memory processes. We show that basic operations such as addition, scalar multiplication, and vector multiplication can be straightforwardly performed by accumulating photon counts. Furthermore, the computation of multiple tasks can be accelerated by mapping them to parallel photon count accumulations. Our experiment also offers a secure method for remote computing, thanks to the probabilistic generation of photons. This ensures that eavesdropping on a small portion of the computation data provides no meaningful information. Additionally, by utilizing correlated photons directly generated within the quantum memory, we demonstrate that the computing speed can be further enhanced, despite the quantum memory’s retrieval efficiency being only around 0.3% (the intrinsic value of 4% multiplied by the total detection efficiency of 7%), which is much lower than the conventional standard for practical quantum memories. These results underscore the quantum-enhanced advantages of in-memory quantum computing and highlight the potential of quantum memories that can be realized with existing technology.

## Results

**Fig. 1:**
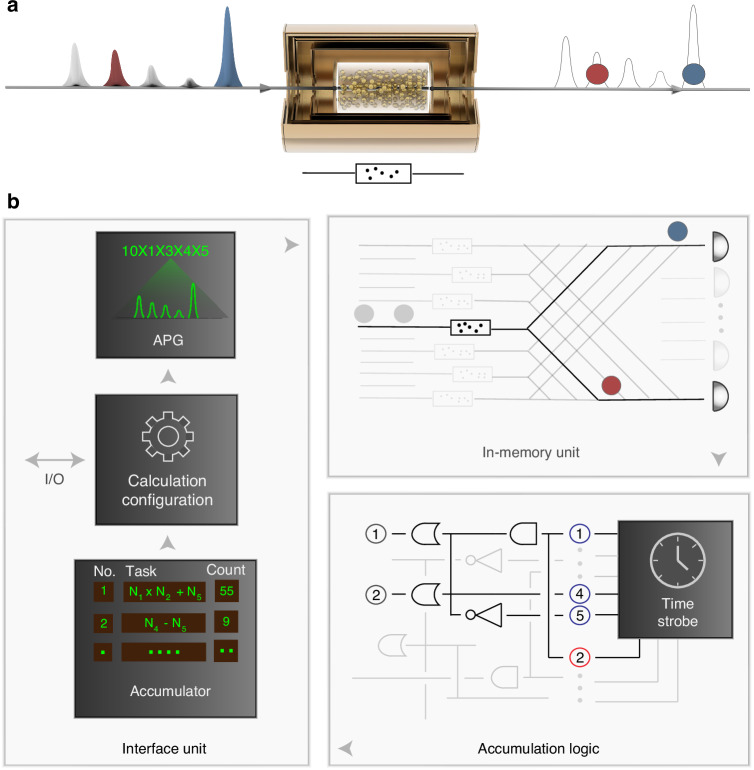
Schematic of in-memory computing based on quantum memory. **a** Quantum memory, as the core of quantum in-memory computing, consists of a caesium atom vapour and a three-layer magnetic shielding. The addressing pulses interact with the atomic ensemble packaged in a glass cell, while generating quantum states, including collective atomic excitations and correlated photons. **b** Quantum enhanced in-memory computing process. Interface unit receives the calculation task and encodes it into the configuration of addressing pulses. In-memory unit converts the analog information of addressing pulses to the generation probability of correlated photons. The output from the single-photon detectors is processed and accumulated according to the accumulation logic designed or programmed by the experimenter. Then the accumulated data (i.e., the detected photon counts) is decoded by the interface unit, and the calculation results can be obtained

**Fig. 2:**
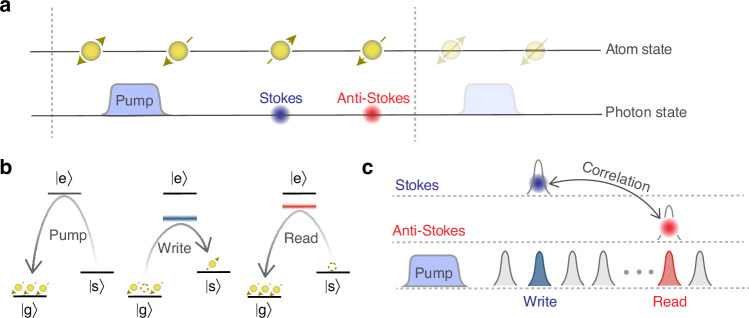
Physical picture of quantum in-memory computing. **a** Time sequence of photons and atomic excitations. Yellow circles in upper line depict atomic state. The blue (red) circle in another line represents the Stokes (anti-Stokes) photon. **b** Physical operations that underpin the in-memory computing. There are three basic operations include pump, write-in and read-out operations. **c** Illustration for a multiplication. A multiplication of two numbers is represented by the joint probability of correlated Stokes photon and anti-Stokes photon. For example, the first (second) number is represented by the detection probability of Stokes (anti-Stokes) photon. A pulse train containing more than two pulses promises to process multiple computation tasks involving additions, scalar multiplications and even vector multiplications in a way of parallel accumulation, see sections SB and SC of Supplementary Information for details

The schematic diagram and physical interpretation of our quantum in-memory computing are shown in Fig. 1 and Fig. 2, respectively. Our in-memory computing is based on a room-temperature quantum memory involving billions of motional atoms, as is shown in Fig. 1a. We illustrate the computing process in Fig. 1b. The computing tasks, such as a computational formula, accepted by the interface unit, are encoded into a corresponding calculation configuration and further into the configuration of addressing pulses. Then, the addressing pulses enter the quantum memory (in-memory unit) and excite or retrieve correlated photons. In this way, the configuration of addressing pulses, as well as calculation tasks, are transferred to the generation probability of Stokes photon, anti-Stokes photon, and atomic spin wave (see Fig. 2). After leaving the quantum memory, the photons generated in a probabilistic manner are detected by single-photon detectors, and the photon counts are accumulated by an accumulator, according to a task-specific accumulation logic. Finally, the interface unit decodes the accumulated data with the information of the calculation configuration, and outputs the calculation results.

In the above calculation procedure, light-matter interaction in the in-memory unit is the key point. Fig. 2a is an illustration of the atomic states and single photons involved in the in-memory unit. To obtain these nonclassical states, three basic physical operations are adopted, as shown in Fig. 2b. A pump light resonant with the transition $| s\rangle \leftrightarrow | e\rangle$ is used to initialize the atoms. After the pump, almost all of the atoms populate the ground state $| g\rangle$. The other two basic physical operations are ‘write in’ and ‘read out’, with which the configuration of addressing pulses is encoded into the generation probability of Stokes photon and anti-Stokes photon. The configuration of these basic operations is task-specific. For example, addition operation can be realized by accumulating photon counts of Stokes photon, thus only the pump operation and write operation are involved. However, multiplication based on the product of two probabilities, is realized by detecting the coincidence of non-classically correlated photons, such as correlated Stokes photons and anti-Stokes photons, as is shown in Fig. 2c.

**Fig. 3:**
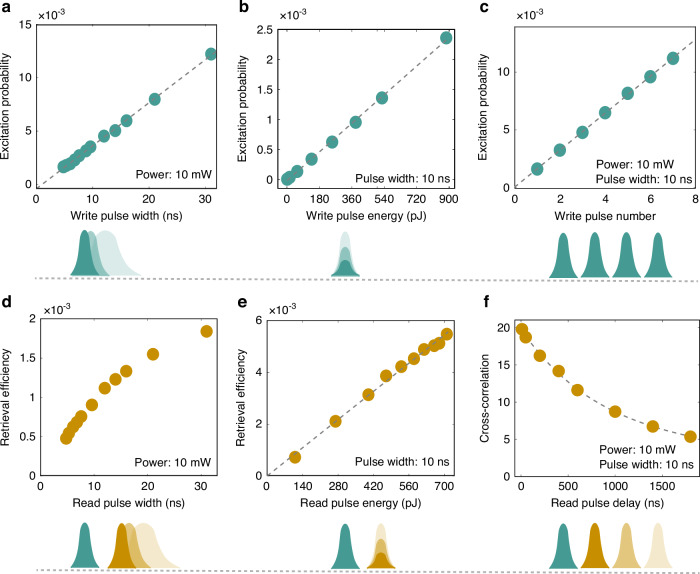
Excitation probability, retrieval efficiency and cross-correlation under different configurations of the addressing pulse. Dark cyan circles represent excitation probability of Stokes photons with different pulse width (**a**), pulse energy (**b**), and number (**c**) of write pulses. Brown circles depict retrieval efficiency of anti-Stokes photons and cross-correlation with different read pulse width (**d**), read pulse energy (**e**), and time delay between write pulse and read pulse (**f**). The error bars are too small to be visible. Dashed grey lines are fitting curves of the experimental data. Dark cyan pulses and brown pulses represent write pulses and read pulses, respectively. The experimental parameters involving light power or pulse width are presented in the bottom right of each sub-figure. The above values of probability and efficiency represent raw data, rather than the intrinsic characteristics of the quantum memory. For instance, the reported retrieval efficiency of 0.3% in the sub-figure (**e**) corresponds to an intrinsic value of 4%, where the total detection efficiency of 7% is taken into account

The quantum memory acts as a time-bin analog-to-digital converter and transfers analog information of the addressing pulses into the generation probability of digital-like information, i.e., single photons. In Fig. 3, we experimentally characterize the property of this analog-to-digital converter under different configurations, such as different settings of pulse width and pulse energy. Figure 3a demonstrates a linear dependence of the excitation probability of Stokes photons on write pulse width, and Fig. 3b shows a linear dependence on write pulse energy. As is shown in Fig. 3c, calculation tasks can be encoded by a bin of write pulses, such as an operation of summing can be encoded on the total generation probability of Stokes photons excited by two or more write pulses. The dependence of retrieval efficiency on the pulse width and pulse energy are shown in Fig. 3d, e, respectively. Figure 3f demonstrates a nonlinear influence of time delay on cross-correlation of Stokes photon and anti-Stokes photon. It is worth mentioning that linear responses of the quantum memory to the addressing pulses can be directly used in a straightforward and reconfigurable computation, while the nonlinear response can be used in some special scenarios, such as the simulation of a nonlinear process^80^.

The Stokes photons and anti-Stokes photons, generated by the addressing pulses in a probabilistic manner, are detected by single-photon detectors. Then the photon counts are recorded by an accumulator. The accumulator is stopped when the accumulated photon counts reach a target count. For obtaining a same target count, different calculation results correspond to different numbers of trials. Therefore, we can obtain the calculation results by recording how many trials are used to achieve the target count. For example, it takes 10,000 trials for accumulating 10 photons, which means a result with a value of 1. Then, the result is 2 when it takes 5000 trials for accumulating 10 photons. However, in practice, there are fluctuations of the number of trials needed to obtain 10 photons, and there is a probability distribution of the number of trials. The width of the distribution is non-zero, which may lead to the overlap between different calculation results. In the overlap, one cannot distinguish different results. For example, the result should be 2, but the trial number may be 10,000 rather than 5000 for obtaining 10 photons under a slightly lower probability, consequently one mistakes this result for 1 under a non-negligible probability.

Both addition and multiplication share a common feature: the operands are encoded in the photon excitation probabilities (for Stokes photons) or the conditional readout efficiencies (for anti-Stokes photons), and the results are obtained by accumulating photon counts. In this sense, the two operations are conceptually similar, as they both rely on counting discrete stochastic events. Specifically, addition corresponds to the classical accumulation of independently generated Stokes photons. Multiplication is realized via the correlation between Stokes and anti-Stokes photons, where the first operand is encoded in the Stokes excitation probability, and the second operand in the conditional readout probability. Mathematically, if ${n}_{i}^{\rm S}$ and ${n}_{i}^{\rm AS}$ respectively denote the *i*-th Stokes and anti-Stokes events (0 or 1), the operations can be expressed as:

$${\bf{Addition:}}\;{N}_{\rm add}=\mathop{\sum }\limits_{i=1}^{M}{n}_{i}^{\rm S},\,\langle {N}_{\rm add}\rangle =M{P}_{\rm S}$$

$${\bf{Multiplication:}}\;{N}_{\rm mult}=\mathop{\sum }\limits_{i=1}^{M}{n}_{i}^{\rm S}{n}_{i}^{\rm AS},\,{P}_{\rm coinc}=\frac{\langle {N}_{\rm mult}\rangle }{M}={P}_{\rm S}{P}_{\rm R}$$

where *P*_S_ is the Stokes photon excitation probability and *P*_R_ the retrieval efficiency. *M* denotes the average trial number required to obtain the photon counts *N*_add_ or *N*_mult_.

**Fig. 4:**
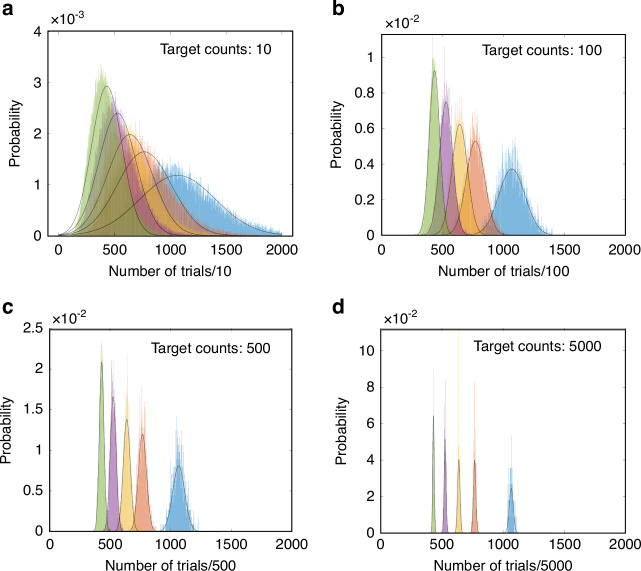
Number of trials required for effectively decoding the accumulated data. The peaks represent the probability distributions of obtaining a target count of Stokes photons, as a function of the number of trials. For example, in (**a**), the probability of obtaining 10 target Stokes photons with 3900 trials is about 0.003, which is the maximum value of green peak. The peaks in different colors correspond to different pulse energy of write pulse, and the peak in green corresponds to the write pulse with the maximum pulse energy, while the peak in blue corresponds to the weakest write pulse. The envelop (black solid line) of each peak is a fitting curve in a form of Gaussian function. (**a**–**d**) correspond to different fixed target photon counts and trial number needed, while the horizontal scale, i.e., the ratio of fixed target photon counts and the corresponding trial number needed, is maintained. The overlap between different peaks decreases with the increasing of target photon counts and trial number, see section SD of Supplementary Information for details

For effectively decoding the accumulated data, i.e., for accurately distinguishing different results, one should carry out enough trials for accumulating enough photon counts, by which the fluctuation and the overlap can be suppressed or eliminated. Figure 4 shows the probability distributions of the trial numbers needed for accumulating a target count of Stokes photons. The peaks in different colors correspond to results with different central values of trial numbers due to the different pulse energies of addressing pulses. From Fig. 4a–d, the overlap between different results decreases with the increasing of target photon count and trial number. Regarding computational accuracy and fidelity, the final result is obtained by summing photon counts, with the target photon number determining the distinguishability of different outcomes. The required target photon number for reliable resolution is analysed in the section SD of the Supplementary Information.

The large overlap in Fig. 4a is an advantage in some sense, since it may secure the computation in a new way. Due to the large overlap, when the photon count is small, no one can eavesdrop an accurate result by eavesdropping a small portion of the photon counts. In a computing network consisting of remote in-memory units and remote accumulators, a secure remote computation may be guaranteed by virtue of this overlap. For example, if large amounts of data are intercepted, eavesdropping will be easily detected. Alternatively, communication is interrupted when the data rate falls below a certain threshold to ensure security. In Fig. 4, the pulse configurations with higher energies appear to correspond to narrower probability distributions. The physical origin of this effect is that a stronger pulse leads to a higher excitation probability and a larger variance for generating Stokes photons. Note that the average number of trials for obtaining one target photon count is, in fact, the inverse of the detected excitation probability.

In quantum communication, security is designed to protect the transmission channel, ensuring that quantum information cannot be intercepted or cloned during propagation (as in quantum key distribution). In contrast, our approach focuses on the protection of the computational process itself. In our system, the computation proceeds through photon-count accumulation within distributed in-memory units and remote accumulators. During this process, the computational state is continuously evolving and has not yet been converted into any readable classical information. An external observer attempting to eavesdrop would only access a highly uncertain quantum state–a mixture of stochastic photon events with overlapping probability distributions–from which no meaningful intermediate results can be inferred. Although the underlying process involves photon exchange and may superficially resemble secure communication, its essential function is to guarantee the confidentiality of quantum computation, akin in spirit to blind quantum computing^81^. Thus, the proposed mechanism should be regarded as a secure quantum computing framework, rather than a communication protocol.

**Fig. 5:**
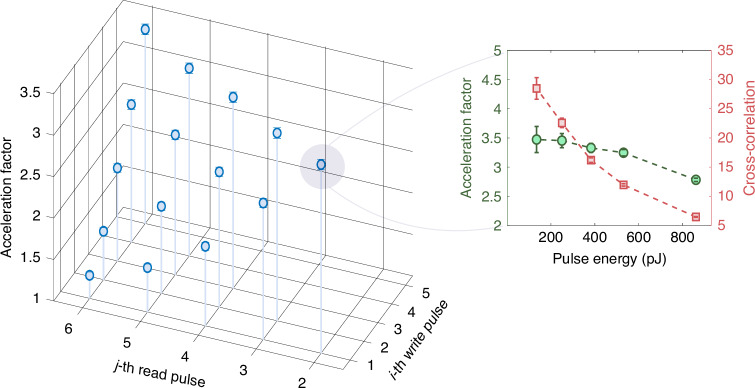
Acceleration factor due to nonclassical correlation. The acceleration factor equals *P*_S*i*−AS*j*_/(*P*_S*i*_*P*_S*j*_). *P*_S*i*_ denotes the probability of Stokes photons generated by the *i*-th write pulse. (*P*_S*i*_*P*_S*j*_) denotes the random coincidence probability of Stokes photons generated by the *i*-th and *j*-th write pulses. *P*_S*i*−AS*j*_ is the joint probability of Stokes photon and anti-Stokes photon, which are respectively excited and retrieved by the *i*-th write pulse and *j*-th read pulse in the same trial. The inset shows the effects of pulse energy on acceleration factor (green circles) and cross-correlation (red squares) when *i* = 1 and *j* = 2

As our in-memory computing is based on the accumulation of photon counts, its computing speed is determined by the generation rate of photons. A high generation rate of photons means a high computing speed. Especially, the rate of coincidence counts of photons from different counting channels (Stokes photon and anti-Stokes photon) is a key parameter for multiplication operations. In our experiment, the coincidence probability between two photons from different trials is lower than the coincidence probability between correlated photons generated in a same trial. For example, when the energy of write and read pulses is 400 pJ, the available retrieval efficiency is around 0.3%, much lower than unit efficiency, but is still larger than the probability (0.1%) of spontaneous Raman scattering in the write process, see Fig. 3b, e. This is the reason why computing speed dependent on the rate of coincidence events can be accelerated by using correlated photons. The acceleration factor (*AF*) is written as the ratio of quantum-enhanced coincidence probability to classically random coincidence probability,

1 $$AF=\frac{{P}_{{\rm S}i-{\rm AS}j}}{{P}_{{\rm S}i}{P}_{{\rm S}j}}$$

where *i* indexes a write pulse and *j* a read pulse. Note that *i* < *j* to ensure that the write pulse is prior to the read pulse. The acceleration factor with different *i* and *j* are shown in Fig. 5. The acceleration factor is about 3 when the Stokes photons and anti-Stokes photons are generated by a pair of adjacent write pulse and read pulse, i.e., *i* = *j* − 1. The acceleration factor reaches its maximum for single-memory operation because the read pulse is applied immediately after the write pulse (i.e., at the minimal delay), leading to the highest read-out efficiency and the strongest temporal correlation between the Stokes and anti-Stokes photons. When multiple read pulses follow a single write pulse, or when the read-out delay increases, the memory coherence and retrieval efficiency decrease. Consequently, *P*_S*i*_*P*_AS*j*_ approaches *P*_S*i*_*P*_S*j*_, and the acceleration effect is diminished.

The inset shows the effects of pulse energy on acceleration factor (green circles) and cross-correlation ${g}^{(2)}=\frac{{P}_{{\rm S}i-{\rm AS}j}}{{P}_{{\rm S}i}{P}_{{\rm AS}j}}$ (red squares) when *i* = 1 and *j* = 2. The acceleration factor, on the other hand, characterises how this nonclassical correlation accelerates the computation under the same excitation probability (i.e., the same pulse energy). A high cross-correlation corresponds to a high acceleration factor, which implies the advantage of nonclassical correlation for in-memory computing. The acceleration factor is maximized when the quantum correlation between the write-in and read-out processes are strongest–namely, in the single-memory, minimal-delay configuration. Therefore, the computing speed scales with both the excitation probability and the strength of the nonclassical correlation: higher excitation increases the photon generation rate, while stronger correlation enhances the effective accumulation rate, together determining the overall acceleration of computation.

## Discussion

The logical operation takes place within the quantum memory through light-matter interaction, where correlated quantum states are generated and stored. The photon-number accumulation during detection merely maps the pre-existing quantum correlations into classical outcomes. Therefore, the operation on the quantum state (via coherent evolution inside the atomic ensemble) is physically distinct from the photon-number accumulation (a classical readout process). The multiplication operation inherently exhibits the key characteristics of in-memory computing. Specifically, during the write process, the joint excitation of a Stokes photon and its correlated atomic excitation encodes the first operand (*A*) in the excitation probability of the Stokes field. During the subsequent read process, a properly tuned read pulse converts the stored atomic excitation into an anti-Stokes photon, with the retrieval probability encoding the second operand (*B*). The coincidence probability between the Stokes and anti-Stokes photons thus represents the product *A* × *B*, realized entirely through light-matter interaction within the same atomic ensemble.

It is worth emphasizing that the quantum in-memory computing system and classical in-memory computing address different computational paradigms–the former focuses on quantum randomness and correlation-enhanced computing, while the latter targets deterministic parallel computation^28^. Keeping this distinction in mind, it is insightful to make some comparisons between the two approaches. In a classical photonic in-memory computing platform based on phase-change materials (PCMs), the crystallization and amorphization processes (i.e., initialization) require optical powers on the order of 5–14 mW, while the energy per write pulse is typically a few hundred picojoules. In the quantum-memory-based in-memory computing system, the optical power required for initializing the atomic ensemble is of the same order (around 10 mW), and the energy of each write/read pulse is also a few hundred picojoules. Therefore, the energy consumed per operation in our system is comparable to that of the classical photonic in-memory computing approach. In addition, there are two key distinctions. First, in the quantum version, the signal photons (Stokes and anti-Stokes photons) are generated intrinsically within the quantum memory, without requiring external signal light input, which leads to an inherent energy-saving advantage compared to classical optical systems that rely on continuous input beams (typically tens of microwatts). Second, the computation demonstrated in this work is a stochastic quantum process based on the generation of truly random single photons, with a typical generation probability of about 1%. Reliable computational outcomes are obtained by accumulating a few hundred photon-counting events. This operating mechanism is fundamentally different from conventional in-memory computing systems, which employ deterministic optical signals.

In summary, we have demonstrated an instance of quantum-enhanced in-memory stochastic computing by mapping computation tasks into two basic physical operations, including the write-in and read-out of a room-temperature quantum memory. Computation is completed through two steps. Firstly, encode the analogue information depicting computation tasks into the generation probability of digital-like photons. Then, after a period of accumulation of the photon counts, the accumulated data is decoded to the desired calculation results according to the target photon counts and used trial number. We analyse the required computing resources for completing addition and multiplication, as well as the corresponding physical processes in quantum memory device. By characterising the encoding and decoding process, we further analyse the performance of in-memory computing enabled by intrinsic randomness and non-classical correlation, which introduces two advantages of quantum in-memory computing: Our results promise a secure way for remote quantum computation^82–84^. The security of computing is guaranteed by quantum randomness, since the calculation results are obtained by accumulating randomly generated photons, and it is hard to infer a precise result based on a small portion of detection events, due to the overlap of probability distribution of needed trial number. Furthermore, in classical regime, in order to increase computing speed, one needs to raise the pulse energy to reach a high generation probability of photons. While in our experiment, non-classical correlation is shown to accelerate computing by higher coincidence probability with a same pulse energy as the classical situation. Sections SE and SF of Supplementary Information provide comprehensive comparisons with the classical in-memory computing systems and bit-stream generators.

In-memory computing is limited in classical regime due to the lack of efforts on exploring the significance of imperfect quantum memories in computing, and it is usually suggested that a quantum memory without a high memory efficiency is impractical for memory-based long-distance and large-scale quantum networks^85,86^, while our results show that a quantum memory with a low memory efficiency can be utilized for constructing quantum-enhanced in-memory stochastic computing. As long as the coincidence probability of correlated photons is higher than that of uncorrelated photons, the computing involving additions and multiplications can be accelerated. Now is perhaps the time to consider the question: Is one imperfect quantum memory practical? Maybe, in addition to further efforts of exploring quantum memories with better performance, some efforts should be dedicated to have a more comprehensive knowledge and a full exploitation of the significance of imperfect quantum memories. A quantum memory that’s not perfect for the generally expected applications may be just right for some others that are usually unnoticed. A notable example, quantum memory hasn’t been fully exploited for constructing unconventional computing in a way of non-deterministic storage.

For real-life applications, the computing speed is dependent on the generation rate of photon counts. Therefore, the improvement of available memory efficiency (including efficiencies in write and read processes) enables the capability of rapidly processing computation tasks. There are many other aspects to further improve the performance of in-memory computing with quantum memory, such as a broad bandwidth memory for data-intensive works and a long-life memory for multiplications of large vectors. Combining with integrated photonic techniques^87–89^ and spatial multiplexing techniques^90–92^, which promise applications in quantum information processing^93,94^, a compact in-memory quantum computing device may be realized. Furthermore, the coherence stored in quantum memory is an engine of quantum computing, which is urgently required in the construction of future in-memory quantum computing. In addition to in-memory computing, the potential applications of an imperfect quantum memory also include light-matter interface for investigating hybrid interference^95,96^ and fundamental tests of quantum mechanics^97^, as the quantum states stored in quantum memory can be nontrivially operated^98^. In a word, our work takes one step towards the in-memory quantum computing, as well as emphasizes the role of imperfect quantum memories in quantum computing and provides enlightenment to future researches on quantum memories.

### Materials and methods

One external cavity diode laser acts as the source of pump light with sensitive temperature and frequency feedback, and provides frequency reference for other lasers. A distributed Bragg reflector laser locked to the reference laser with a frequency difference of 4 GHz is used as the source of write/read pulses. The continuous wave from the distributed Bragg reflector laser is chopped by an Electro Optic Modulator (EOM). Then, a tapered amplifier is utilized to boost the power of write/read pulses. Cesium atoms are packed in a 75 mm-long cylindrical glass cell with 10-Torr Ne buffer gas. The glass cell is placed in a three-layer magnetic shielding, and is heated up to 61 ^∘^C. Before entering cesium cell, addressing pulses are horizontally polarized by a Glan-Taylor polarizer. The polarization of generated Stokes photons and anti-Stokes photons are vertical to that of write/read pulses, due to which we can use a Wollaston prism to basically filter out the write/read pulses. A frequency filter, consisting of six home-made cascaded cavities, separates the Stokes photons from anti-Stokes photons, and filters out the noise photons. The transmission rate of every cavity is higher than 90%, and the extinction ratio of every cavity is up to 500:1. Stokes photons and anti-Stokes photons are detected by different single-photon detectors, and the detected photon counts are recorded by a multi-channel counting system for further processing.

For realizing scalar multiplication, coincidence counts between correlated Stokes photons and anti-Stokes photons are used. For realizing vector multiplication, which is a fundamental operation for matrix manipulation, multiple pairs of write pulse and read pulse should be implemented. For example, $[{N}_{1}\,\,{N}_{2}\,\,{N}_{3}]{[{M}_{1}\,\,{M}_{2}\,\,{M}_{3}]}^{T}$ can be realized by three pairs of write pulse and read pulse. The first pair of pulses encodes the product of *N*_1_ and *M*_1_, and the second pair encodes *N*_2_*M*_2_. Based on the ability of completing addition and multiplication in a predetermined accumulation logic, an acceleration is brought into the calculation process because of the intrinsic parallel computing mode. Each write operation probabilistically generates a correlated pair consisting of a Stokes photon and an atomic spin excitation through spontaneous Raman scattering. The intrinsic excitation probability per write pulse is typically around 1% (or even lower), meaning that, on average, only one successful excitation occurs in about one hundred trials. Consequently, different write processes are almost statistically independent, and no coherent interference between successive write pulses is expected. This provides a key strength of our approach: it inherently mitigates the accumulation of computational errors as the vector size increases. Based on our experimental observations, if one write operation successfully creates an atomic excitation and a Stokes photon, the probability of generating another excitation in the subsequent write pulse increases slightly–by approximately 0.5%. This minor effect arises from enhanced Raman scattering due to the pre-existing atomic excitation and can be compensated by a slight adjustment of the write-pulse energy. The stochastic and low-probability nature of the Raman write process ensures that no phase control across pulses is required, and quantum-state interference between different write-in pulses does not occur under our experimental conditions.

## Supplementary information

Supplementary Materials

## Data Availability

All study data are included in the article and/or supporting information.
