# Supplementary material for: Quantum-enhanced reconfigurable in-memory stochastic computing
Source: Light Sci Appl. 2026 Mar 18;15:178. doi: 10.1038/s41377-025-02181-6 (PMC13000280; doi:10.1038/s41377-025-02181-6)
Supplement: Supplementary file 1 — Supplementary Materials [file 41377_2025_2181_MOESM1_ESM.pdf]

Supplementary Information for Quantum-enhanced reconfigurable in-memory stochastic computing

Hong-Zhe Yang[1,2,†], Jian-Peng Dou[1,2,†], Feng Lu[1,2], Xiao-Wen Shang[1,2], Chao-Ni Zhang[1,2], Heng Zhou[1,2], Hao Tang[1,2], Xian-Min Jin[1,2,3,4*]

[1]Center for Integrated Quantum Information Technologies (IQIT), School of Physics and Astronomy and State Key Laboratory of Photonics and Communications, Shanghai Jiao Tong University, Shanghai 200240, China

[2]Hefei National Laboratory, Hefei 230088, China

[3]TuringQ Co., Ltd., Shanghai 200240, China

[4]Chip Hub for Integrated Photonics Xplore (CHIPX), Shanghai Jiao Tong University, Wuxi 214000, China

*E-mail: xianmin.jin@sjtu.edu.cn

†These authors contributed equally to this work

SA Foundational concepts and challenges in stochastic computing, in-memory computing, and quantum computing

SA.1 Stochastic computing (SC)

Stochastic computing (SC) is a computational paradigm that encodes real-valued numbers as probabilistic bitstreams [1, 2]. In SC, the value of a number is encoded in the probability of a bit being '1' in a stream of binary bits. For example, a value of 0.75 might be represented by a bitstream in which 75% of the bits are '1' and the remaining 25% are '0'. Unlike traditional binary arithmetic, SC operates by processing these bitstreams directly, performing operations like addition and multiplication through bitwise operations that rely on statistical averages.

In SC, the main computational operations, such as addition and multiplication, are performed on the bitstreams:

(1) Addition is accomplished by performing a weighted sum of two bitstreams, where the number of ‘1’ bits in each stream represents the numerical value.

(2) Multiplication involves combining two bitstreams using bitwise logical operations, often producing a stream that represents the statistical product of the two original numbers.

(3) Complex operations can be implemented by combining these basic operations, though SC becomes less efficient for more complex calculations due to increased error rates and slower convergence for higher precision.

SC has several advantages, such as its low hardware complexity and natural tolerance to noise, which makes it suitable for applications in embedded systems, low-power computing, and signal processing. However, its accuracy is directly tied to the length of the bitstream, and achieving higher precision requires longer streams, which results in slower computation. Additionally, SC faces challenges in performing complex operations efficiently and scaling to large datasets, due to the need for extended bitstream lengths. In addition, SC relies on the generation of random-biased bitstreams to represent real-valued numbers. Typically, these random bitstreams are generated using pseudo-random number generators (PRNGs), which, despite being widely used, incur a computational cost. Generating high-quality (pseudo-)random bits can be significantly more expensive than implementing basic arithmetic operations, such as a full adder. As a result, the gate-level advantages of stochastic computing—particularly in terms of hardware simplicity and low power consumption—are often diminished, as the overhead of generating random bits can negate the benefits of using SC for certain applications.

However, **one of the fundamental features of quantum memory is its inherent ability to produce truly random outcomes, which can be harnessed to generate controllably biased photon streams suitable for stochastic computing applications.** Quantum systems, by their nature, exploit the probabilistic behavior of quantum states, making them ideal for generating random bitstreams with high entropy. For example, quantum memory techniques such as light-matter interaction and atomic spin-wave storage inherently produce random results due to the

uncertainty principle and quantum measurement processes.

By utilizing quantum memory to generate random photon streams, one can bypass the costly process of using pseudo-random number generators in SC systems. This could significantly reduce the computational overhead associated with random bit generation, thus restoring or even enhancing the gate-level advantage of SC. The random photon streams generated through quantum memory would offer both high-quality randomness and a direct source of entropy, potentially enabling more efficient and faster stochastic computations. This approach could also provide a more robust method of randomness, addressing some of the limitations of classical PRNGs, such as predictability and bias in large-scale computations. Thus, **by combining quantum memory with stochastic computing, we may be able to harness the power of quantum randomness to improve the efficiency and feasibility of SC, making it more competitive for a wider range of applications.**

SA.2 In-memory computing (IMC)

In-memory computing (IMC) refers to computational models where data is processed directly within memory storage units, rather than being transferred to a central processing unit (CPU) for computation. This model significantly reduces data transfer delays, which are often a bottleneck in traditional computing systems, and can lead to improved processing speeds and energy efficiency.

IMC leverages novel memory technologies, such as resistive RAM (ReRAM) and phase-change memory (PCM), enabling computation to occur directly within the memory cells. The challenge, however, lies in scaling these memory technologies for large, complex applications, as well as integrating them with existing computational infrastructures. IMC also faces difficulties related to data integrity, fault tolerance, and managing large amounts of data while maintaining high throughput. Recent research has focused on developing hybrid systems that combine the strengths of IMC with other architectures, like stochastic computing, to create

energy-efficient, high-performance computational systems. Yet, these efforts are still in the early stages, and practical implementations are limited by issues such as hardware instability and limited scalability.

SA.3 Quantum computing (QC)

Quantum computing (QC) exploits the principles of quantum mechanics, such as superposition, entanglement, and interference, to process information in fundamentally different ways compared to classical computing. Quantum algorithms have shown potential to solve certain problems exponentially faster than classical counterparts [3, 4, 5], such as factoring large numbers or simulating quantum systems.

Quantum computers rely on quantum bits, or qubits, which can exist in multiple states simultaneously due to superposition. Additionally, qubits can be entangled, meaning the state of one qubit can influence another, even at great distances. These properties allow quantum computers to perform certain calculations more efficiently than classical computers. However, there are significant challenges to overcome, including:

(1). Qubits are highly sensitive to their environment, and maintaining their coherence long enough to perform calculations is a major hurdle.

(2). Quantum systems are prone to errors, and efficient quantum error correction methods are still under development.

(3). Current quantum hardware is limited in terms of the number of qubits and the connectivity between them, which constrains the ability to implement large-scale quantum algorithms.

Despite these challenges, ongoing research in quantum error correction, qubit design, and hybrid quantum-classical systems is steadily moving the field toward practical applications.

SA.4 Integration and future directions

Integrating SC, IMC, and QC presents a promising avenue for overcoming the limitations of each paradigm. For example, SC's natural tolerance to noise and its ability to process proba-

bilistic information could complement quantum computing's parallelism and in-memory computing's high throughput. Specifically:

(1) SC's probabilistic nature could benefit from quantum algorithms that handle uncertainty and superposition, improving precision and efficiency in computations.

(2) IMC can provide the low-latency data storage and processing required for quantum and stochastic operations, potentially leading to hybrid systems that can support large-scale, energy-efficient quantum computations.

(3) Combining these paradigms could address key challenges in scaling quantum systems, as the noise-resilient characteristics of SC and the rapid data processing capabilities of IMC may help mitigate some of the difficulties related to quantum decoherence and error rates.

The integration of these three computational models is still in its early stages, and much research is needed to overcome the technical hurdles associated with combining quantum mechanics, probabilistic computing, and memory-centric architectures. However, the potential for high-impact applications in fields such as machine learning, optimization, and real-time data processing makes this an exciting area for future exploration.

SB Details of experimental setup.

One external cavity diode laser acts as the source of pump light with sensitive temperature and frequency feedback, and provides frequency reference for other lasers. A distributed Bragg reflector laser locked to the reference laser with a frequency difference of 4 GHz is used as the source of write/read pulses. The continuous wave from the distributed Bragg reflector laser is chopped by an Electro Optic Modulator (EOM). Then, a tapered amplifier is utilized to boost the power of addressing pulses. Cesium atoms are packed in a 75mm-long cylindrical glass cell with 10-Torr Ne buffer gas. The glass cell is placed in a three-layer magnetic shielding, and is heated up to 61 °C. Before entering cesium cell, addressing pulses are horizontally polarized by a Glan-Taylor polarizer. The polarization of generated Stokes photons and anti-Stokes photons

are vertical to that of addressing pulses, due to which we can use a Wollaston prism to basically filter out the addressing pulses. A frequency filter, consisting of six home-made cascaded cavities, separates the Stokes photons from anti-Stokes photons, and filters out the noise photons. The transmission rate of every cavity is higher than 90%, and the extinction ratio of every cavity is up to 500:1. Stokes photons and anti-Stokes photons are detected by different single-photon detectors, and the detected photon counts are recorded by a multi-channel counting system for further processing.

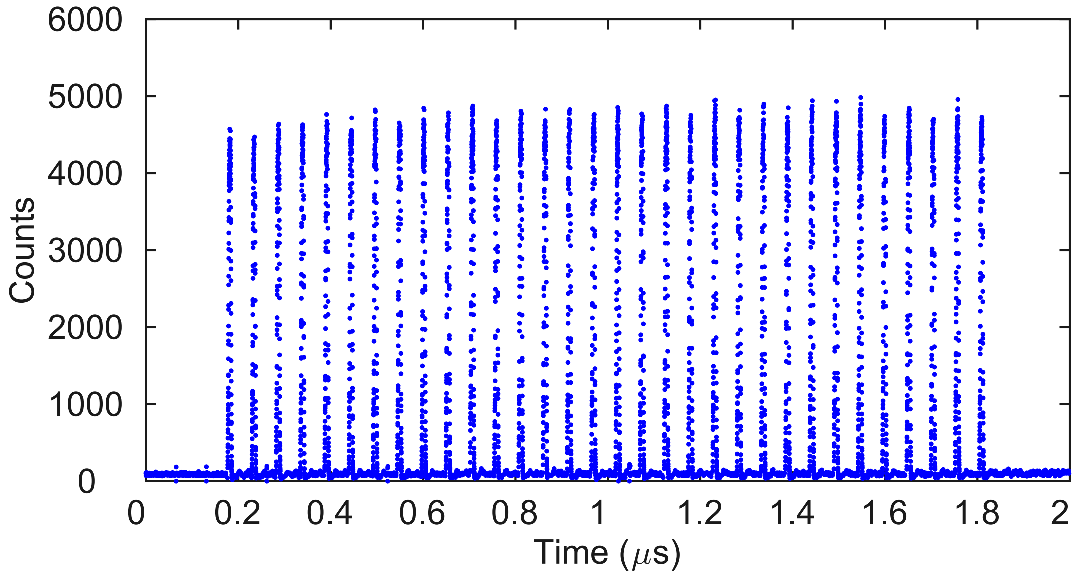

Figure S1: **Stokes photon counts in 32 time sites.** The number of trials for each peak is 500000.

Each write operation probabilistically generates a correlated pair consisting of a Stokes photon and an atomic spin excitation through spontaneous Raman scattering. The intrinsic excitation probability per write pulse is typically around 1% (or even lower), meaning that, on average, only one successful excitation occurs in about one hundred trials. Consequently, different write processes are almost statistically independent, and no coherent interference between successive write pulses is expected. This provides a key strength of our approach: it inherently mitigates the accumulation of computational errors as the vector size increases. As shown in Fig. S1, we measured the Stokes photon count distribution across 32 time modes. The results

demonstrate that the photon count waveform is nearly uniform at each time node.

Based on our experimental observations, if one write operation successfully creates an atomic excitation and a Stokes photon, the probability of generating another excitation in the subsequent write pulse increases slightly–by approximately 0.5%. This minor effect arises from enhanced Raman scattering due to the pre-existing atomic excitation and can be compensated by a slight adjustment of the write-pulse energy. The stochastic and low-probability nature of the Raman write process ensures that no phase control across pulses is required, and quantum-state interference between different write-in pulses does not occur under our experimental conditions.

In addition, due to the finite storage lifetime, the ground-state population gradually decreases over time (i.e., polarization relaxation), which leads to a lower excitation probability in later write processes. To maintain stable operation, we periodically apply a pump beam (10mW) from the external cavity diode laser to reinitialize the atomic ensemble into its ground state.

Furthermore, our scheme is inherently scalable. Since the computational pulses are time-multiplexed and propagate through the same optical path, they experience nearly identical losses and noise. This common-mode effect allows these errors to be normalized out in the processing stage. Consequently, even pulses separated by long time intervals do not exhibit significant relative errors. Other potential issues, such as those arising from laser power and frequency jitter or slow thermal drift in frequency filters, are related to specific device imperfections. We consider these to be technical challenges that can be effectively optimized in the future with improved engineering and control systems, and they do not represent a fundamental limitation to the scalability of the scheme's core principle.

SC Accumulation rule for parallel computation.

In our experiment, the light-matter interface acts as a random photon source. The computing task, such as $N_1 - N_2 + N_3 - N_4$ where N_i ($i = 1, 2, 3, 4$) represents an arbitrary number, is encoded on addressing pulses in chronological order. Number N_i is encoded on the energy of

one addressing pulse, and the corresponding relation between them is linear. For example, number 1 is encoded on an addressing pulse with pulse energy E. Then, number 2 corresponds to pulse energy $2E$. After the interaction between the addressing pulse with energy E and atoms, Stokes photons are detected with a probability of p, while an addressing pulse with energy $2E$ corresponds to a probability of $2p$. Therefore, the sum of numbers N_i is transformed to the sum of probabilities $p_1 - p_2 + p_3 - p_4$. Specific accumulation rules are used to retrieve the probability value over a period of accumulation. For example, the counts of Stokes photons generated by the first and third addressing pulses add up in the accumulator. And the photon counts corresponding to the second and fourth addressing pulses are subtracted. For computation tasks which have a same element, such as $N_1 + N_3$, $N_1 + N_2$ and $N_1 + N_2 + N_3$, the photon counts for number N_1 contribute to all probabilities of $p_1 + p_3$, $p_1 + p_2$ and $p_1 + p_2 + p_3$. Therefore, parallel computing is realized.

For realizing scalar multiplication, coincidence counts between correlated Stokes photons and anti-Stokes photons are used. For realizing vector multiplication, which is a fundamental operation for matrix manipulation, multiple pairs of write pulse and read pulse should be implemented. For example, $[N_1 \;\; N_2 \;\; N_3][M_1 \;\; M_2 \;\; M_3]^T$ can be realized by three pairs of write pulse and read pulse. The first pair of pulses encodes the product of N_1 and M_1, and the second pair encodes $N_2 M_2$. Based on the ability of completing addition and multiplication in a predetermined accumulation logic, an acceleration is brought into the calculation process because of the intrinsic parallel computing mode.

SD Derivation of formulas in calculation process.

As is shown in Fig. 4 in the main text, the envelope of each peak is a fitting curve in the form of Gaussian function

$$\frac{1}{\sqrt{2\pi}\sigma}e^{-\frac{(x-\mu)^2}{2\sigma^2}}, \tag{S1}$$

where x denotes the average number of trials for obtaining one target photon. The Gaussian function can be fully determined by the standard deviation σ and the central value μ, which respectively reflect the width of the Gaussian function and correspond to the position for the maximum value of the Gaussian function.

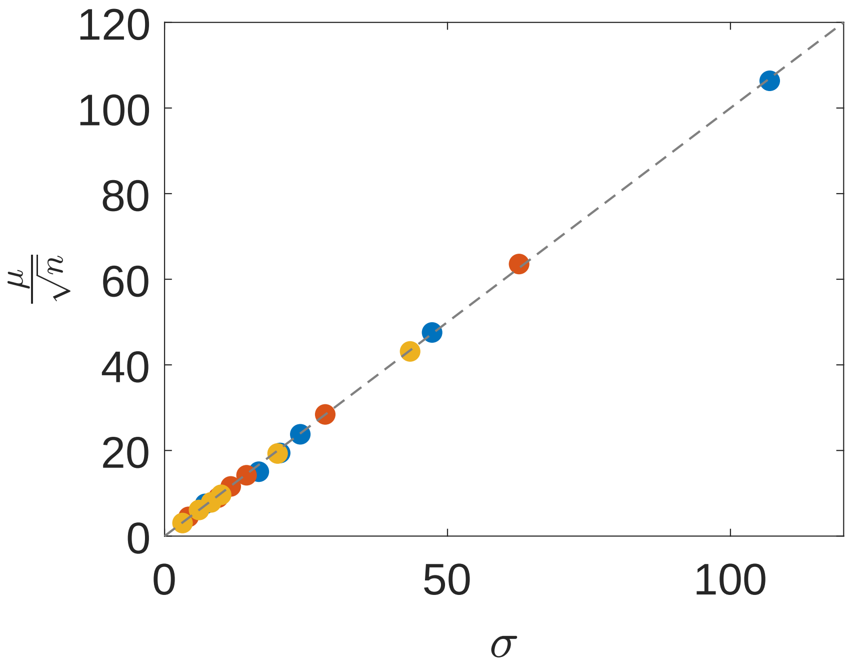

Figure S2: **Fitting the relation between standard deviation σ and $\frac{\mu}{\sqrt{n}}$.** σ is the standard deviation of Gaussian function Eq. (S1). μ is the central coordinate of the Gaussian function. n denotes target photon count. The dashed gray line represents fitting function with formation $\frac{\mu}{\sqrt{n}} = c\sigma$. And the fitting parameter c is 1.001. Circles in different colors (yellow, blue and red) represent different settings of pulse energy. Note that, a fixed pulse energy corresponds to a fixed excitation probability p of photons, and thus corresponds to a fixed central value μ no matter what the target photon count n is, while a different pulse energy corresponds to a different central value μ.

As shown in Fig. S2, by fitting σ and μ with different target counts n, we obtain the following

relation between these three parameters.

$$\sigma = \frac{\mu}{\sqrt{n}}. \tag{S2}$$

In an interval ranging from $\mu - 3\sigma$ to $\mu + 3\sigma$, more than 99% of the total probability (i.e. the area under the envelope of any one peak in Fig. 4) is contained, based on which we use the following formula to determine the target count for ensuring negligible overlap between adjacent peaks.

$$\mu_2 - \mu_1 = 3(\frac{\mu_2}{\sqrt{n}} + \frac{\mu_1}{\sqrt{n}}), \tag{S3}$$

$$n = 9(\frac{\mu_1 + \mu_2}{\mu_1 - \mu_2})^2, \tag{S4}$$

where μ_1 is the central coordinate of leftmost peak which corresponds to maximum calculation result, while μ_2 is the central coordinate of the second peak from left. The reason why we use μ_1 and μ_2 is that the leftmost peak and its adjacent peak become distinguishable in the last with raising target count, as shown in Fig. 4 in the main text.

The central value μ, representing the average number of trials for obtaining one target count, is in fact the inverse of the detected excitation probability p of Stokes photons.

$$\mu = \frac{1}{p}. \tag{S5}$$

According to Fig. 3 of main text, the linear relation between the pulse energy and excitation probability can be written as the following

$$p = KNE_{\min}, \tag{S6}$$

$$E_{\min} = \frac{E_{\max}}{N_{\max}}, \tag{S7}$$

where K is the coefficient of mapping a specific pulse energy to an excitation probability of Stokes photons, and N is an arbitrary number to be encoded. $E_{\min}$ represents the minimum pulse energy can be used to encode computation tasks. For example, the number $N = 1$ is

encoded by a pulse with pulse energy of E_{min}. Similarly, the number $N = 10$ is encoded by $10E_{\text{min}}$ corresponding to an excitation probability $10KE_{\text{min}}$. N_{max} is the maximum number that can be encoded on one pulse, and E_{max} is the highest energy of one pulse.

If multiple pulses are implemented in each cycle, and the photon counts stemmed from all implemented pulses are accumulated as a total photon count, then the maximum number that can be encoded is lN_{max} instead of N_{max}, where l is the total number of pulses implemented in each cycle. The second largest number that can be encoded is $lN_{\text{max}} - 1$. By substituting $N = lN_{\text{max}}$ and $N = lN_{\text{max}} - 1$ into Eq. (S6), one can obtain μ_1 and μ_2 respectively

$$\mu_1 = \frac{1}{p} = \frac{1}{K(lN_{\text{max}})E_{\text{min}}}, \tag{S8}$$

$$\mu_2 = \frac{1}{p} = \frac{1}{K(lN_{\text{max}} - 1)E_{\text{min}}}, \tag{S9}$$

Correspondingly, in Eq. (S4), the target count, needed for ensuring that adjacent peaks centered at μ_1 and μ_2 become distinguishable, can be written as

$$n = 9(2lN_{\text{max}} - 1)^2. \tag{S10}$$

Eq. (S10) suggests that the leftmost peak and its adjacent peak, respectively corresponding to the largest encoded number $N = lN_{\text{max}}$ and second largest encoded number $N = lN_{\text{max}} - 1$, become distinguishable in the last with raising target photon count. Because n depends on N_{max}, any number N less than N_{max} corresponds to a smaller n. That is, for distinguishing two adjacent peaks with larger encoded numbers N, one needs a larger target photon count n than that of peaks with smaller encoded numbers N.

Based on Eq. (S10), the time required for completing computation tasks can be obtained

$$N_{\text{try}} p_{\text{min}} = n, \tag{S11}$$

$$t_{\text{total}} = N_{\text{try}} T_{\text{try}}, \tag{S12}$$

where N_{try} is the number of trials needed for achieving the target photon count n by using a minimum excitation probability $p_{\text{min}} = KE_{\text{min}}$. T_{try} is the time cost for one trial. By combining

Eqs. (S11) and (S12), the total time cost can be written as

$$t_{\text{total}} = \frac{nT_{\text{try}}}{p_{\min}}. \tag{S13}$$

Assume that $T_{\text{try}} = al + b$, where a is the time interval between adjacent addressing pulses, and b is the time cost for initializing the system. Then the total time cost can be expressed as

$$\begin{aligned} t_{\text{total}} &= \frac{[9(2lN_{\max} - 1)^2](al + b)}{p_{\min}} \\ &\approx \frac{72aN_{\max}^2 l^3}{p_{\min}} \\ &= \frac{72aN_{\max}^3 l^3}{KE_{\max}}. \end{aligned} \tag{S14}$$

The above approximation is valid when $2lN_{\max} \gg 1$ and $al \approx b$. Eq. (S14) shows that, with a fixed $N_{\max}$, the time cost scales with l^3. While in a conventional computing architecture, the time cost may grow exponentially with the problem size l, such as the subset sum problem (SSP). Therefore, an acceleration may be achieved with our in-memory computing in dealing with some complex problems intractable for conventional computers.

SE Comparison with classical in-memory computing systems

To date, a memory system that operates intrinsically in the nonclassical regime has not been explored for demonstrating in-memory computing. Indeed, our current work provides a principle demonstration of the concept of using quantum storage-generated bit-streams for in-memory computing, focusing primarily on the qualitative aspects of the approach, such as the inherent randomness of the quantum process and the potential advantages of quantum correlations for specific operations. For now, we hope that the qualitative insights we present help establish a solid foundation for further exploration of this exciting new direction. While such a comparison is certainly valuable, there are three key characteristics of our system that distinguish it from classical in-memory computing:

(1) Non-classical correlations arising from quantum memory operations: Our system leverages quantum memory for both writing and reading operations, which introduces unique non-classical correlations that classical systems cannot replicate. These quantum correlations enable a fundamentally different computational paradigm, where the quantum memory itself contributes to the computation in ways that go beyond classical in-memory systems.

(2) Quantum memory provides a unique advantage, as it generates true randomness, notably through phenomena such as Stokes photon emission during the write process. These photons exhibit true quantum randomness, which is fundamentally different from the pseudo-random numbers produced by classical methods. This inherent randomness allows for the generation of bit-streams without the need for complex correlation control mechanisms, typically associated with classical pseudo-random number generation.

(3) Probabilistic photon generation leading to stochastic computing: The probabilistic nature of photon generation in our system introduces a stochastic aspect to the computation. This randomness is a key feature of our approach and provides a unique advantage in certain applications, particularly in secure remote computing, where the probabilistic nature of photon generation ensures that no meaningful information can be gained by eavesdropping on a small portion of the data.

While classical in-memory computing systems excel in scalability and efficiency for many tasks, our approach offers unique quantum-enhanced features that could open up new avenues for computation, especially in applications that benefit from quantum randomness and correlations. This could provide advantages in fields such as secure computing, optimization, and real-time processing, where classical systems may face limitations. Table SI below provides a comparison with classical in-memory computing systems.

Table SI: **Comparison between quantum in-memory computing and classical in-memory computing.**

	Classical in-memory computing	Quantum in-memory computing
Quantum correlation	No	Yes
True randomness	No	Yes
Scalability	Yes	Yes
Secure remote computing	No	Yes
Parallelism	Yes	Yes
Ability to do stochastic computing	Yes	Yes
Number of operation steps for generating bit streams	3 steps:<br>(1) Encoding the material's transmittance by light of electrical pulse;<br>(2) Injecting the optical or electrical signal;<br>(3) Detecting the output signal.	2 steps:<br>(1) Encoding pulse energy;<br>(2) Detecting the output signal.

SF Comparison with CMOS-based generators

In our work, the SC bit-streams are generated through the interaction of light with atomic ensembles (specifically, using quantum memory in the form of spin-wave storage). During the write process, Stokes photons are produced through the interaction of the control field with the atomic ensemble. These Stokes photons exhibit true randomness due to the quantum nature of the system. After the photons are generated, the bit-streams are generated by the detection of

these Stokes photons. The read process involves retrieving the information from the quantum memory, and anti-Stokes photons are emitted. These photons, which are correlated with the Stokes photons, are then detected and converted into bit-streams.

This process ensures that the generated bit-streams maintain the inherent quantum randomness of the system, distinguishing them from pseudo-random streams produced by classical methods. This feature can be particularly valuable in applications requiring high-quality randomness, such as cryptographic systems and high-precision computational tasks. However, several challenges remain:

Scalability: One of the primary challenges is scalability. While quantum memory is effective at generating bit-streams at small scales, scaling this approach to larger systems remains technically demanding. The integration of quantum memory with conventional computing hardware may require significant advancements in quantum memory and control systems.

Efficiency: The process of generating quantum bit-streams, particularly through atomic memory, may be slower than conventional CMOS-based generators, especially for applications requiring high throughput. This tradeoff between speed and quality will need to be carefully considered for different applications.

Table SII below provides a comparison with CMOS-based generators.

In our work, we focus on the non-classical correlations between the Stokes photons emitted during the write process and the anti-Stokes photons retrieved during the read process. These correlations are another key feature of our approach and distinguish our quantum-generated bit-streams from those produced by classical pseudo-random number generators. Regarding the allocation of bit-streams to time bins, our work incorporates a reconfigurable approach that allows for the dynamic distribution of bit-streams across multiple time bins. Specifically, we use a series of addressing pulses, modulated by electro-optic devices, to control the energy distribution between the write and read pulses. This enables us to dynamically adjust the retrieval efficiency of the anti-Stokes photons and thus the allocation of bit-streams to different time bins.

Table SII: **Comparison between quantum memory-based generators and CMOS-based generators.**

	Quantum memory-based generator	CMOS-based generator
Generation method	Quantum processes, such as photon emission in quantum memory systems.	Classical noise sources (e.g., thermal noise, shot noise) amplified and processed.
Randomness quality	True randomness based on quantum mechanics, ideal for cryptography and high-security applications.	Pseudo-random, determined by the noise source and circuit design, may not meet high-security standards.
Technology maturity	Cutting-edge, still under development and research; not widely accessible.	Highly mature, widely used, and integrated into many electronic devices.
Power consumption	Generally higher, especially with cryogenic setups or complex quantum devices.	Extremely power-efficient due to CMOS technology.
Generation speed	Slower due to the complexity of quantum state preparation and measurement.	Faster, capable of generating millions of bits per second.
Applications	High-security cryptography, quantum key distribution (QKD), and quantum computing.	Embedded systems, consumer electronics, low-cost applications, and general random number generation.

References

[1] Alaghi, A., Qian, W. & Hayes, J. P. The promise and challenge of stochastic computing. *IEEE Transactions on Computer-Aided Design of Integrated Circuits and Systems* **37**, 1515-1531 (2018).

[2] Daniels, M. W., Madhavan, A., Talatchian, P., Mizrahi A. & Stiles, M. D. Energy-Efficient

Stochastic Computing with Superparamagnetic Tunnel Junctions. *Phys. Rev. Applied* **13**, 034016 (2020).

[3] Google Quantum AI and Collaborators. Quantum error correction below the surface code threshold. *Nature* **638**, 920-926 (2025).

[4] Deng, Y.-H. *et al.* Gaussian Boson sampling with pseudo-photon-number-resolving detectors and quantum computational advantage. *Phys. Rev. Lett.* **131**, 150601 (2023).

[5] AbuGhanem, M. Photonic quantum computers. https://doi.org/10.48550/arXiv.2409.08229 (2024).
